# STABILITY AND CHANGE IN SOCIAL ISOLATION PROFILES OVER TIME AMONG OLDER ADULTS

**DOI:** 10.1093/geroni/igad104.1384

**Published:** 2023-12-21

**Authors:** Pildoo Sung, Angelique Chan

**Affiliations:** Hong Kong Baptist University, Hong Kong, Hong Kong; Duke-NUS Medical School, Singapore, Singapore

## Abstract

This study examined transitions in social isolation profiles over time and factors associated with them, using longitudinal data from 1,305 older adults in Singapore. While prior studies have investigated social isolation profiles that combine objective measures of social disconnectedness and subjective feelings of loneliness, less is known about the stability and change in these profiles over time. We utilized random-intercept latent transition analysis to identify social isolation profiles and their transition patterns over time. Multivariable regression then examined whether health and sociodemographic characteristics were associated with specific transitions of interest. The empirical analyses yielded three key findings. First, at baseline, about 10% of older adults were “disconnected and lonely,” while 30% were “disconnected but less lonely.” The remaining respondents were “connected and less lonely.” Second, at follow-up, 44% retained their baseline profiles, while 56% transitioned into different profiles. Third, older adults with lower socioeconomic status were more likely to transition from the “connected and less lonely” profiles to the disconnected and less lonely profile. Older adults with declining mental health were more likely to remain in or transition into the “disconnected and lonely” profile. In summary, approximately half of the older Singaporeans were socially disconnected, and one in five of them perceived loneliness occasionally or more often. Socioeconomically disadvantaged older adults were more likely to experience social disconnectedness over time, but this does not necessarily accompany loneliness. Rather, it is declining mental health that leads to both social isolation and loneliness, which calls for tailored interventions.

